# Analysis of IVIM Perfusion Fraction Improves Detection of Pancreatic Ductal Adenocarcinoma

**DOI:** 10.3390/diagnostics14060571

**Published:** 2024-03-07

**Authors:** Katarzyna Nadolska, Agnieszka Białecka, Elżbieta Zawada, Wojciech Kazimierczak, Zbigniew Serafin

**Affiliations:** Collegium Medicum, Nicolaus Copernicus University in Torun, Jagiellońska 13-15, 85-067 Bydgoszcz, Polandserafin@cm.umk.pl (Z.S.)

**Keywords:** pancreatic ductal adenocarcinoma, intravoxel incoherent motion, diffusion-weighted imaging, perfusion-dependent diffusion coefficient, diagnostic accuracy

## Abstract

The purpose of this study was to evaluate whether intravoxel incoherent motion (IVIM) parameters can enhance the diagnostic performance of MRI in differentiating normal pancreatic parenchyma from solid pancreatic adenocarcinomas. This study included 113 participants: 66 patients diagnosed with pancreatic adenocarcinoma and 47 healthy volunteers. An MRI was conducted at 1.5 T MR unit, using nine b-values. Postprocessing involved analyzing both conventional monoexponential apparent diffusion coefficient (ADC) and IVIM parameters (diffusion coefficient D-pure molecular diffusion coefficient, perfusion-dependent diffusion coefficient D*-pseudodiffusion coeffitient, and perfusion fraction coefficient (f)) across four different b-value selections. Significantly higher parameters were found in the control group when using high b-values for the pure diffusion analysis and all b-values for the monoexponential analysis. Conversely, in the study group, the parameters were affected by low b-values. Most parameters could differentiate between normal and cancerous tissue, with D* showing the highest diagnostic performance (AUC 98–100%). A marked decrease in perfusion in the patients with pancreatic cancer, indicated by the significant differences in the D* medians between groups, was found. In conclusion, standard ADC maps alone may not suffice for a definitive pancreatic cancer diagnosis, and incorporating IVIM into MRI protocols is recommended, as the reduced tissue perfusion detected by the IVIM parameters is a promising marker for pancreatic adenocarcinoma.

## 1. Introduction

Pancreatic ductal adenocarcinoma (PDAC) is the most common primary malignant neoplasm of the pancreas, occurring in approximately 80% of cases [1]. PDAC is usually fatal disease, typically affecting the elderly or people older than 55 years of age, with the highest incidence in the 7th and 8th decades of one’s life [2,3]. Only 20% of patients with this diagnosis can undergo radical surgical treatment [4,5], and the tumor’s stage at diagnosis remains the most significant prognostic factor [5]. The mean 5-year survival for PDAC, according to the literature, ranges from 2 to 9% [4,6].

On imaging, PDAC is usually visible as a moderately hypovascular mass within the pancreatic parenchyma [7]. Therefore, cross-sectional modalities present limited diagnostic accuracy in the early detection of this malignancy. While CT presents a sensitivity of 90% and a specificity of 99% for detecting solid pancreatic neoplasms, the sensitivity sharply decreases to 77% for lesions smaller than 2 cm [8,9]. In a study which compared three screening modalities in 225 high-risk individuals, endoscopic ultrasound (EUS) detected pancreatic abnormalities in 43% of the subjects, MRI in 33%, and CT in 11% of them. These abnormalities included suspected or proven neoplasms in 85 cases, including intraductal papillary mucinous neoplasm (IPMN) in 82 of them, and neuroendocrine tumors in three patients [8]. Moreover, chronic pathologies of the pancreas, including distortions after acute inflammation, chronic inflammation, and autoimmune pancreatitis, may hamper the diagnosis [10]. In fact, only complications of PDAC result in a clear diagnosis using cross-sectional imaging. These include pancreatic duct obstruction, infiltration in adjacent structures, and, less often, nodal involvement.

Although the general concept of contrast enhancement in CT and conventional MRI is similar, MRI still has a higher tissue resolution, which makes it a better option for the imaging of small or CT iso-attenuating masses [11,12]. However, MRI can also offer a functional option, namely, diffusion-weighted imaging (DWI), which presents the motion of water molecules within tissues. The DWI signal is composed of both microperfusion and diffusion within the extracellular space. Restriction of water diffusion increases the DWI signal. This restriction may result from several pathological processes, including cellular swelling, an increase in cellular density, and desmoplastic reactions. Conventional DWI has many applications, mostly in neuroimaging and oncology. DWI has already shown a promising potential in predicting the malignant transformation of intraductal papillary mucinous neoplasms of the pancreas [13]. However, in the case of PDAC, the advantage of DWI is not obvious. 

A more advanced analysis of the DWI signal, called intravoxel incoherent motion (IVIM) imaging, has been proposed by Le Bihan [14]. IVIM, using several b-values in the DWI sequence, allows for the separation of pure diffusion signal and diffusion signal related to microperfusion (or pseudo-diffusion), which can be described using the following measures: isolated diffusion coefficient (D), perfusion-dependent diffusion coefficient (D*), and perfusion coefficient (f) [14]. This method has been gaining increasing recognition in recent years, especially in the imaging of oncological diseases [15]. IVIM perfusion MRI enables the imaging of neoangiogenesis and microcirculation heterogeneity and the monitoring of the effects of radio- or chemotherapy treatments or antiangiogenetic drugs [16,17,18,19,20]. However, there are mixed reports regarding its value in differentiating between metastatic and non-metastatic lymph nodes [21,22]. The use of the IVIM MRI method in the differentiation of chronic pancreatitis [23] and autoimmune pancreatitis from pancreatic cancer [24] has been demonstrated. Since PDAC, compared to normal pancreatic parenchyma, presents a moderate hypoperfusion on CT scans and conventional MRIs, IVIM has the potential to increase the visibility of this type of tumor. 

The aim of this study was to prove the hypothesis that the analysis of IVIM parameters increases the value of MRI in differentiating between normal pancreatic parenchyma and solid adenocarcinomas.

## 2. Materials and Methods

### 2.1. Material

This study included 113 subjects. The study group consisted of 66 patients aged 41–89 years who had been referred for surgery due to pathologically proven PDAC (mean BMI 26.3 kg/m^2^). The inclusion criteria were a histopathological diagnosis of PDAC and informed consent to participate in this study. The control group consisted of 47 healthy volunteers aged from 25 to 63 years (mean BMI 23.5 kg/m^2^). In both groups, the exclusion criteria were the following: pregnancy, below 18 years of age, and routine contraindications to MRIs (claustrophobia, or foreign bodies incompatible with the MR environment).

### 2.2. Magnetic Resonance Imaging and Postprocessing

Examinations were performed using a 1.5 T unit (Optima 450wGEM, GE Healthcare, Milwaukee, WI, USA) with an eight-channel abdominal surface coil in the supine position, with respiratory triggering. To localize the anatomical structures, T2-weighted imaging was performed in sequences of fast spin echo with fast relaxation (FRFSE) in a breath-hold in the frontal and transverse planes with fat saturation, with the following parameters: TR, 4.3 ms; TE, 100 ms; layer thickness, 5 mm; layer spacing, 0.5 mm; field of view (FOV), 33 cm; and matrix, 320 × 224. T1-weighted breath-hold imaging was also performed in the transverse plane with fat saturation using, with the following parameters: TR, 135 ms; TE, 100 ms; thickness layers, 5 mm; layer spacing, 0.5 mm; FOV, 33 cm; and matrix, 224 × 320. Diffusion sequences were generated in the transverse plane using the planar echo technique. The diffusion sequence described as IVIM had nine different b-values (0, 10, 20, 50, 100, 200, 400, 600, and 1000 s/mm^2^), with a layer thickness of 6 mm and a layer spacing of 1 mm. 

Postprocessing of the diffusion signal in the four sets of b-values presented in Table 1 consisted of measuring the signal (i) in the monoexponential (standard) model, which was the apparent diffusion coefficient (ADC), and (ii) in the biexponential model, namely, the IVIM (D*, D, f). In addition, signal intensity in T2-weighted images was measured to standardize image quality. The measurements were performed using a dedicated workstation Olea Sphere 3.0 (Olea Medical, La Ciotat, France). The oval regions of interest (ROI) with an average size of 91 pixels (158.5 mm^2^) were positioned in the center of the tumor mass (omitting foci of necrosis and cystic areas) in the participants in the study group. In the control group, the ROI was positioned in the lower part of the head of the pancreas, outside the main pancreatic duct.

### 2.3. Statistical Analysis

The qualitative variables were characterized using the frequency of occurrence of a given subcategory. The quantitative variables were initially assessed for normal distribution using the Kolmogorov–Smirnov and Shapiro–Wilk tests. Depending on the type of distribution, the quantitative variables were described using the mean or median and standard deviation or upper and lower quartiles. Student’s *t*-test or the non-parametric Mann–Whitney test were used to compare two dependent variables, and, for the independent variables, the Levene homogeneity of variance test and the *t*-test of equality of means were used. Repeated measures ANOVA was used to compare more than two variables. Alternatively, for non-normally distributed variables, the Friedman test was used to compare the medians of multiple dependent variables. Post hoc pairwise comparisons were then performed. The relationship between the quantitative and qualitative variables was assessed on the basis of Pearson’s correlations and non-parametric Spearman’s rho correlations. The statistical significance of the differences between the measurement results in the study and control groups was assessed using the Mann–Whitney test. If significant differences were found, the value of the technique was tested by an ROC analysis. The assumed significance level for the verification of the statistical hypotheses was 0.05. These analyses were performed using the MedCalc v. 18 package (MedCalc Software bvba, Amsterdam, The Netherlands).

## 3. Results

In the literature, several sets of b-values have been used. Therefore, no standard protocol for IVIM postprocessing has been established. A comparison of the coefficients of variation of the tested parameters in the control group is shown in Table 2. Ratios above 50% were reported for ADC2, D3, D*1, D*2, D*4, and f2. For these parameters, one can discuss their clinical usefulness or logarithmic transformation usage.

**Figure 1:**
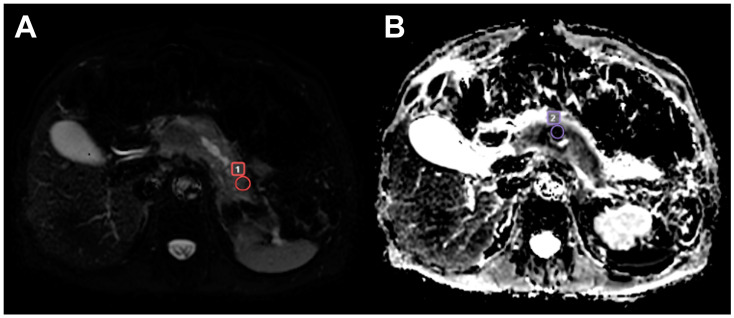
ROI placement in a bifocal ductal adenocarcinoma of the pancreas: (**A**) a DWI image at b0 with an ROI in the tail tumor (1); and (**B**) an ADC image with an ROI in the trunk tumor (2).

In the study group, the mean tumor diameter was 42.3 mm (range between 13 and 89 mm). Staging of the disease was not considered in this study. Figure 1 presents a sample ROI positioning of a patient with bifocal PDAC. 

**Figure 2:**
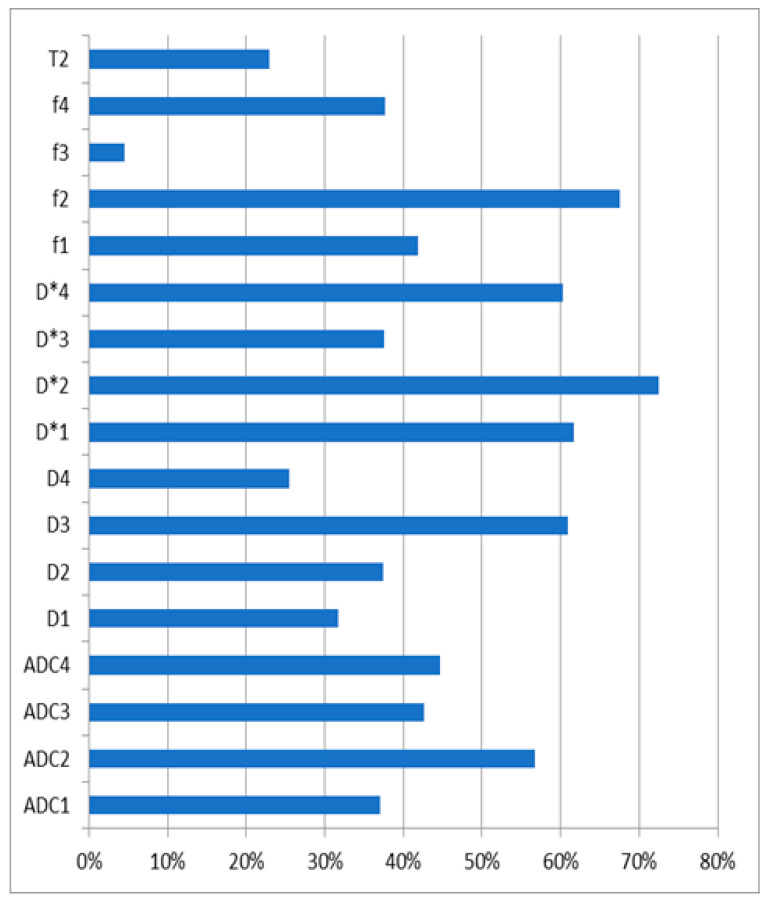
A comparison of the coefficients of variation of the tested parameters in the control group.

A comparison of the measurement results in the control and study groups is presented in Table 2. Significant differences were observed in all the parameters, except for D4, f3, and T2. A similar result was found in the AUC analysis (Table 3). Interestingly, all the D* measures had an AUC value of 1.0. The graph presenting the comparison of the coefficients of the tested parameters in the control group can be found in Figure 2.

## 4. Discussion

This paper presents a prospective single-center clinical trial on patients diagnosed with pancreatic cancer and a control group. In both study groups, the IVIM parameters were dependent on the set of b-values used for the calculations. In general, it was shown that, in the control group, the IVIM parameters were significantly higher for the set of high b-values resulting from the pure diffusion biexponential analysis and for the monoexponential analysis of all b-values. Regardless of the b-value set, the perfusion-dependent diffusion coefficient (D*) showed an excellent diagnostic value, with an AUC of 100%.

Recent studies have emphasized the dependence of perfusion in pancreatic tissue images on the MRI methodology. The analysis of changes in the pancreas, i.e., PDAC, neuroendocrine neoplasms, acute and autoimmune inflammations, and pseudopapillary tumors which are characterized by a different histological structure, pays more attention to the interpretation of parameters in the biexponential model [25,26]. Most recent studies have emphasized the role of perfusion f-fraction in the differentiation between PDAC and normal pancreatic parenchyma [25,27]. It has been shown that the f-fraction of perfusion depends on the histological density of vessels [28]. This has been confirmed in research works as a significant difference in the perfusion fraction (f) of neuroendocrine tumors and PDCA [26,27]. Neuroendocrine tumors are characterized by a rich network of capillaries, whereas PDCA is hypovascular and characterized by a high desmoplastic reaction with a low microvascular density. Similar relationships have been demonstrated for D/D* [26,27,28,29], which describe the collective movement of flowing water molecules inside capillaries. In a study by Robertis et al. [26], the perfusion fraction achieved 100% sensitivity in differentiating PDAC from the normal pancreas and autoimmune inflammation from normal pancreatic tissue. The specificity for D and f was higher than 80% in the cases involving the differentiation of PDAC from neuroendocrine tumors and for the differentiation of neuroendocrine tumors from autoimmune pancreatitis [26]. However, a study by Chao Ma et al. [29] showed that diffusion parameters (in the mono- and biexponential model) were not useful for assessing PDCA and did not differ in different tumor locations (in the pancreas or in metastases).

Several studies have focused on differentiating autoimmune pancreatitis (AIP) from PDAC. AIP is a special type of inflammation characterized by a large fibrous reaction with dense glandular tissues and inflammation. If fibrous changes affect a large part of the gland, the MR image resembles PDAC and may be confused with the malignancy. Considering the completely different treatment and prognosis of these two conditions, proper diagnosis is crucial. A correct diagnosis protects patients with AIP from unnecessary surgery and protects patients with PDAC from delayed diagnosis and steroid therapy. Klauβ et al. [24] conducted a study on a group of 15 patients with AIP, 11 healthy patients, and 20 patients with PDAC. They performed diffusion tests using the IVIM method with eight b-values, from 50 to 800 s/mm^2^. They showed significantly lower perfusion fraction values in AIP than in PDAC. 

Kim et al. [27] conducted a retrospective study in which the study group consisted of 60 patients with PDAC, 15 patients with neuroendocrine tumors, 9 patients with solid pseudopapillary tumor, and 30 patients with pancreatitis; the control group consisted of 30 subjects. Magnetic resonance imaging was performed using the IVIM technique with 10 b-values (0–900 s/mm^2^). The study showed that the perfusion parameters f and D* were most helpful in characterizing PDAC. The authors emphasized the dependence of these parameters on the degree of tumor vascularity. Their study showed the possibility of differentiating a highly vascularized pancreatic neuroendocrine tumor from PDAC (rich in fibrous tissue) using f. A meta-analysis by Zhu et al. on the diffusion imaging of pancreatic tumors reported 31 papers including 1558 patients with pancreatic pathologies [30]. The results confirmed that DWI and IVIM enable a good differentiation between malignant and benign lesions of the pancreas. They emphasize the importance of ADC and f measurements being the most accurate. They also showed that 3 T MR units present a higher sensitivity of ADC and a higher specificity of f compared to 1.5 T machines [30]. These results are different from ours. However, the above-mentioned meta-analysis was designed as a large study including patients with different pancreatic pathologies. Such a large number of cases must have resulted in a significant heterogeneity of the data. In addition, the IVIM acquisition parameters were not standardized, which may have led to different measurement results in different studies.

The present study has several limitations. First, our study and control groups had a limited size; therefore, our results require confirmation in a larger population. Second, we tested only several sets of b-values to calculate the IVIM measures. However, we believe that it is the time to reach a consensus on the optimal MRI protocol to use and set multi-organ standards to use the capabilities of IVIM most effectively. Third, differences in age and BMI between the study and control groups should be emphasized. We did not consider these differences; however, in further studies, groups should be fitted more precisely to limit possible doubts regarding results. Finally, the study design did not consider possible differences in the histological structure of the pancreatic tissue. In the study group, it might have been the content of fibrous tissues (desmoplastic reaction) to affect the obtained measurements, while, in the control group, it might have been the amount of interlobular adipose tissue. 

## 5. Conclusions

In conclusion, we found IVIM to be a very promising tool to significantly increase the diagnostic accuracy of MRI in detecting PDAC. The diffusion-dependent coefficient (D*) seems to be of special importance for further studies. Simultaneously, IVIM postprocessing is becoming increasingly available in commercially offered workstations and is not time-consuming. We would like to advise the use of multi-b-value DWI sequences in the routine MR imaging of pancreatic tumors. 

## Figures and Tables

**Table 1:** Analyzed sets of b-values.

Set	Parameters	Applied b-Values [s/mm^2^]
1.	ADC1, D*1, D1, f1	all: 0, 10, 20, 50, 100, 200, 400, 600, 1000
2.	ADC2, D*2, D2, f2	low: 0, 10, 20, 50, 100, 200
3.	ADC3, D*3, D3, f3	high: 400, 600, 1000
4.	ADC4, D*4, D4, f4	0 and high: 0, 400, 600, 1000

ADC—apparent diffusion coefficient; D*—perfusion-dependent diffusion coefficient; D—pure molecular diffusion coefficient; f—perfusion fraction coefficient (f).

**Table 2:** Comparison of measurement results in the control group and in the study group. The mean values and their 95% confidence intervals are given.

Parameter	Control Group Mean (95% CI)	Study Group Mean (95% CI)	Mean Difference	*p*-Value
ADC1	1.86 (1.66–2.06)	1.24 (1.15–1.33)	0.62	<0.0001
ADC2	2.03 (1.69–2.36)	1.34 (1.25–1.42)	0.52	<0.0001
ADC3	1.96 (1.71–2.20)	1.41 (1.34–1.48)	0.55	<0.0001
ADC4	2.51 (1.47–3.54)	1.60 (1.40–1.79)	0.91	0.0054
D*1	18.5 (15.1–21.8)	12.4 (9.4–15.4)	6.04	0.0080
D*2	17.7 (14.0–21.5)	9.16 (7.78–10.54)	8.56	0.0001
D*3	55.7 (49.5–61.8)	30.9 (25.7–36.1)	24.79	0.0001
D*4	19.2 (15.8–23.0)	8.08 (6.78–9.39)	11.11	0.0001
D1	1.19 (1.08–1.30)	1.01 (0.94–1.08)	0.18	0.0054
D2	2.88 (2.56–3.20)	1.64 (1.49–1.79)	1.24	0.0001
D3	1.78 (1.46–2.10)	1.10 (1.02–1.18)	0.67	0.0001
D4	1.18 (1.10–1.27)	1.17 (1.09–1.25)	0.01	0.8118
f1	0.40 (0.35–0.45)	0.27 (0.23–0.31)	0.13	0.0001
f2	0.23 (0.19–0.28)	0.10 (0.09–0.12)	0.13	0.0001
f3	0.97 (0.96–0.99)	0.95 (0.90–0.99)	0.03	0.3072
f4	0.37 (0.33–0.41)	0.20 (0.16–0.23)	0.17	0.0001
T2	676 (627–724)	640 (609–672)	36.5	0.1981

ADC1–4, actual diffusion coefficients; D*1–4, perfusion-dependent diffusion coefficients; D1–4, coefficients of isolated diffusion; f1–4, perfusion diffusion fraction coefficients; and T2, transverse relaxation time.

**Table 3:** Comparison of the measurement results in the control group and in the study group. The AUC values, their 95% confidence intervals, the cut-off thresholds, the sensitivity and specificity of the methods at the calculated cut-off thresholds, and the statistical significance of the calculations are provided.

Parameter	AUC (95% CI)	Sensitivity	Specificity	Threshold	*p*-Value
ADC1	0.78 (0.69–0.86)	93%	53%	≤1.65	<0.0001
ADC2	0.76 (0.66–0.84)	84%	66%	≤1.48	<0.0001
ADC3	0.79 (0.70–0.87)	71%	81%	≤1.46	<0.0001
ADC4	0.75 (0.53–0.90)	89%	71%	≤1.86	0.0711
D*1	1.00 (0.97–1.00)	100%	100%	≤1.57	<0.0001
D*2	1.00 (0.97–1.00)	100%	100%	≤3.22	<0.0001
D*3	1.00 (0.97–1.00)	100%	100%	≤1.84	<0.0001
D*4	1.00 (0.97–1.00)	100%	100%	≤1.67	<0.0001
D1	0.64 (0.53–0.73)	82%	51%	≤1.19	0.0163
D2	0.86 (0.78–0.92)	89%	72%	≤2.21	<0.0001
D3	0.76 (0.66–0.84)	71%	77%	≤1.18	<0.0001
D4	0.51 (0.41–0.61)	70%	38%	≤1.3	0.8777
f1	0.71 (0.61–0.79)	84%	51%	≤0.41	<0.0001
f2	0.84 (0.75–0.90)	63%	89%	≤0.10	<0.0001
f3	0.51 (0.41–0.61)	93%	2%	>0.95	0.931
f4	0.82 (0.73–0.89)	70%	83%	≤0.24	<0.0001
T2	0.59 (0.49–0.69)	43%	81%	≤592	0.1347

ADC1–4, actual diffusion coefficients; D*1–4, perfusion-dependent diffusion coefficients; D1–4, coefficients of isolated diffusion; f1–4, perfusion diffusion fraction coefficients; and T2, transverse relaxation time.

## Data Availability

The data associated with our study are confidential due to the nature of medical records and imaging studies; however, access might be granted in justified cases. To gain access, please contact the corresponding author.
